# Parental Autonomy Support and Psychological Well-Being in Tibetan and Han Emerging Adults: A Serial Multiple Mediation Model

**DOI:** 10.3389/fpsyg.2019.00621

**Published:** 2019-03-21

**Authors:** Xiaoyu Lan, Chunhua Ma, Rendy Radin

**Affiliations:** ^1^Faculty of Psychology, Beijing Normal University, Beijing, China; ^2^Department of Developmental Psychology and Socialization, University of Padova, Padua, Italy; ^3^Department of Psychology, Northwest Minzu University, Lanzhou, China; ^4^FISPPA, University of Padova, Padua, Italy

**Keywords:** psychological well-being, parental autonomy support, growth mindset, grit, Tibetan emerging adult

## Abstract

A growing body of research has explored well-being in diverse cultural contexts, and indicates that the definition and perception of well-being vary according to cultural context. Little is known, however, about whether intercultural differences in China (i.e., Tibetan and Han) lead to different perceptions of well-being and how social contexts and personal characteristics are associated with well-being in Tibetan and Han emerging adults. Using a self-determination framework, the current study examines the relationship between parental autonomy support (PAS) and psychological well-being (PWB) in Tibetan and Han emerging adults in China. Guided by implicit theory and self-regulatory theory, we propose a serial multiple mediation model of growth mindset and grit in the association between PAS and PWB. Propensity score matching was used to balance the two ethnic groups in terms of age, gender, socioeconomic status (SES), with a ratio of one to two. Finally, 59 Tibetan (71.2% girls) and 118 Han (69.5% girls) emerging adults aged from 18 to 25 years were included in the current study, and completed an online questionnaire survey. Findings suggest that (a) Tibetan emerging adults perceived higher levels of PWB than their peers from the Han ethnic group; (b) a serial multiple mediation model for the association between PAS and PWB was supported in Han emerging adults; (c) the indirect effects between PAS and PWB varied between Tibetan and Han emerging adults. Our findings suggest that PAS and grit contribute to PWB of emerging adults in both cultural contexts, whereas growth mindset may be beneficial for Han emerging adults only.

## Introduction

Abundant studies have ascertained the antecedents and outcomes of psychological well-being (PWB) in recent decades (e.g., see a meta-analysis by Sin and Lyubomirsky, 2009), but little is known about intercultural psychological perspectives of well-being since the conception and perception of well-being vary by different cultural contexts (Sachs et al., 2018). As a typical collective society, China is well-known for diverse intercultural differences, which are mainly shaped by indigenous Chinese philosophy (i.e., Confucianism, Daoism) and Buddhism (Lu, 2005; Tang, 2015), however, these philosophical thoughts and cultural traditions have distinct views of human happiness. Influenced by Buddhism, Tibet emphasizes mental cultivation and spiritual enlightenment, whereas on the basis of Confucianism, the Han ethnic group (the majority ethnic group in China) attaches more importance to social harmony and collective welfare (Lu, 2005). The Tibetan and Han ethnic groups are rooted in different philosophical thoughts and cultural traditions, and it is still unclear from empirical research whether the perception of PWB between Tibetan and Han emerging adults is distinct.

Relatively little is known about how to facilitate levels of PWB from a self-determination perspective, especially in a collective society, which is crucial to the psychosocial development of emerging adults in both Tibetan and Han populations. Indeed, whether satisfaction in the need for autonomy facilitates the PWB of emerging adults in a collective setting is still under debate (Vansteenkiste et al., 2005). Importantly, few studies have examined the underlying mechanisms between autonomy support and PWB, which would deepen our knowledge of the association between autonomy support and psychosocial development in emerging adults from different cultural contexts.

According to a framework from self-determination theory (SDT), autonomy as a basic human need is critical to well-being (Ryan and Deci, 2000). SDT has further identified autonomy-support as a parenting practice that improves children’s intrinsic life goals, which in turn enhance psychosocial functioning (Chirkov and Ryan, 2001). Intrinsic life goals are pursued as an expression of inherent growth tendencies (Kasser and Ryan, 1996), enabling individuals to pursue their goals despite difficulties. From this perspective, individuals who believe basic attributes are malleable and can be developed or cultivated through effort, are more likely to make sustained efforts, which in turn may enhance well-being for individuals. Guided by implicit theory and self-regulatory theory (Dweck et al., 1995; Pintrich, 2004), we propose a serial multiple mediation model of growth mindset (i.e., the malleability of basic attributes) and grit (i.e., aspirations for long-term interests and goals) to explain the underlying mechanism between parental autonomy support (PAS) and PWB.

From a theoretical perspective, the current study contributes considerably to the literature, in particular to the literature about intercultural differences in PWB and to shed light on psychosocial functioning in Tibetan emerging adults. From a practical perspective, determining how PAS affects PWB through growth mindset and grit is crucial to understanding how social contexts and individual characteristics influence well-being in both cultural contexts, which will direct educators and practitioners to create culture-based intervention programs to improve the level of PWB.

### Psychological Well-Being for Tibetan and Han Emerging Adults

Emerging adulthood is a new conception of development, with a focus on ages between 18 and 25 (Arnett, 2000). There are several common transitions in life during this period, such as commitment to intimate relationships, job seeking or the pursuit of higher education (Arnett, 2000). In the Chinese cultural context, the stressful challenges during emerging adulthood are often amplified due to the longstanding link between academic success and cultural values (Quach et al., 2015). This potentially vulnerable group faces a number of burdens and challenges, and so the current study investigates the correlates of PWB in Tibetan and Han emerging adults.

Psychological well-being refers to the emotional and cognitive evaluation of the quality of life (Ryff, 1995), and can be conceptualized through different approaches, specifically *hedonia* and *eudaimonia* (Deci and Ryan, 2008). The former usually refers to the experience of positive emotional states and the satisfaction of desires that a person currently experiences, whereas the latter involves the state of human potential, such as the presence of meaning and the development of one’s potentials (Disabato et al., 2016). Since Chinese cultures attach more importance to social harmony and interrelationships (Huang, 2012), previous study has indicated that the notion of eudaimonic well-being is more acceptable to Chinese values (Tang et al., 2016). In view of this, the current study defines well-being from an eudaimonic perspective and assesses it accordingly.

Informed by a perspective of culture and well-being, PWB can be defined in universal terms, but also needs to be understood within the framework of individual cultures (Oishi and Diener, 2003; Tov and Diener, 2009). In China, Confucianism, Buddhism and Daoism form the backbone of Chinese culture and each has distinct views on human happiness (Lu, 2005; Tang, 2015). In consideration with cultural entities, the current study compares PWB in Tibetan and Han cultural contexts. On the basis of different cultural beliefs, Tibet’s cultural values are deeply rooted in Buddhism. From a Buddhist perspective, happiness can be defined as a state of flourishing that arises from mental balance and insight into the nature of reality, rather than a fleeting emotion or mood aroused by sensory and conceptual stimuli (Ekman et al., 2005; Wallace and Shapiro, 2006). On the other hand, Han is greatly influenced by Confucianism, underlining social harmony and social interactions (Tang, 2015). As such, happiness involves the establishment and maintenance of interpersonal harmony and the growth of wealth and welfare in groups (e.g., family) (Tseng and Hsu, 2018). As they are also strongly influenced by Western cultural values, Chinese youth are going through drastic social transformations in which competition for wealth and social resources are becoming fierce (Wang, 2006), especially in the Han areas due to the geographical factors. Due to the intercultural differences between Tibetans and Han, we propose that each ethnic group perceives or defines PWB in different ways, and the conditions for PWB in both ethnic groups may differ.

Despite the various cultural differences between Tibetans and Han, both cultural contexts share similar specific cultural values. For example, the conception of PWB in both cultures emphasizes a meaningful life, and harmony in family relations, but not a good mood. From this perspective and informed by SDT (Ryan and Deci, 2000), we propose that autonomy support from parents is positively associated with PWB among Tibetan and Han emerging adults.

### Parental Autonomy Support and Psychological Well-Being

According to SDT (Ryan and Deci, 2000), autonomy is one of the basic psychological needs that contributes to optimal development and functioning, such as greater academic achievement and better psychological health (see a meta-analysis by Vasquez et al., 2016). Research has shown that autonomy in emerging adults could be enhanced by parental support (e.g., Inguglia et al., 2015). PAS refers to parents’ promotion of the increasing demands of emerging adults for independence, such as demands for freedom of expression, thinking and decision-making (Soenens et al., 2007).

Abundant studies focusing on Western cultural contexts have shown that PAS is positively associated with psychosocial adjustment in emerging adults. For example, empirical research showed that autonomy support in close relationships is an essential correlate of PWB (e.g., Demir et al., 2011). Similarly, research shows that PAS is positively associated with higher levels of PWB in Belgian emerging adults (Kins et al., 2009). Interestingly, a cross-cultural study showed that PAS is related to PWB in Chinese and North American adolescents (Lekes et al., 2010), suggesting that PAS is also beneficial to individuals from a collective setting. Additionally, informed by a recent meta-analysis, the strength of the PAS relationship is stronger when PAS is reflective of both parents, rather than of just mothers or just fathers (Vasquez et al., 2016). Accepting this view, the current study focuses on autonomy support from both parents.

Although the association between PAS and PWB is supported by several studies in Western cultures, little is known about the benefits of PAS in a collective society, or the underlying mechanism between PAS and PWB. Culture can impact the PWB process through self-regulatory mechanisms, determining how people think, feel and behave in the pursuit of PWB (Lu, 2005). As such, guided by implicit theory and self-regulatory theory (Dweck et al., 1995; Pintrich, 2004), we propose that growth mindset and grit may serve as serial multiple mediators between PAS and PWB.

### The Mediating Role of Growth Mindset

Growth mindset refers to the belief that an individual’s basic attributes are malleable and can be changed through effort (Dweck et al., 1995). According to the Confucian and Buddhist framework, the perception of growth mindset (e.g., self and the general world) is also distinct due to their different philosophical thoughts and cultural traditions. In terms of selfhood, Confucian thoughts pay special attention to self-cultivation and personal growth through endeavors, whereas Buddhism denies the reality of self because life is viewed as a condition of inherent degradation and there is no life outside the domain of transmigration (Ho, 1995). From the other side, under the influence of Buddhism, Tibetans emphasize spirituality. The Buddhist notion of emptiness (*Kong*) advocates letting be and acceptance instead of active coping, which mitigates human agency (Sundararajan, 2008).

Previous study has found that socialization factors such as autonomy support from parents are positively associated with growth mindset among Chinese children (Kim et al., 2017), suggesting that the quality of autonomy support in a family enables individuals to believe that basic attributes can be improved, such as the general world and personality. Empirical evidence suggests that a high level of growth mindset is positively associated with well-being among adolescents (Kern et al., 2015). Similarly, research also shows that there is a positive association between growth mindset and PWB in Chinese adolescents, indicating that the malleability of a person’s basic attributes can enhance their level of PWB (Zeng et al., 2016).

Although a growing body of empirical research supports the association between PAS and growth mindset, little is known about the connection between the malleability of basic attributes and PWB in emerging adults.

### Grow Mindset and Grit

From a theoretical perspective, implicit theory (Dweck et al., 1995) points out that individuals who believe that attributes are more dynamic, malleable and developable, tend to better understand actions and outcomes concerning more specific psychological mediators. A growing body of empirical research has examined the relations between growth mindset and grit. For instance, a recent meta-analytic review showed that growth mindset positively predicts distinct self-regulatory processes (Burnette et al., 2013). An empirical study from China showed that growth mindset is positively and significantly associated with grit in late adolescents (Wang et al., 2018). Similarly, a recent study exploring the underlying mechanism between growth mindset and grit confirmed a significant main effect wherein growth mindset is positively associated with grit in Chinese adolescents (Zhao et al., 2018). In line with these findings, individuals who believe basic attributes are malleable may make continuous efforts toward goals in spite of setbacks and difficulties. We thus propose a serial multiple mediation model instead of parallel/multiple mediators in the current study. That is, grit may serve as a mediator between growth mindset and PWB in Tibetan and Han emerging adults.

### The Mediating Role of Grit

Grit is defined as a personality trait involving perseverance and passion for long-term goals in the face of adversity (Duckworth et al., 2007). There is still debate about the validity and function of grit (see a review by Credé et al., 2017). For instance, extensive evidence about self-regulatory traits showed that grit is highly correlated with others, such as conscientiousness and self-control (e.g., Duckworth and Gross, 2014), however, research has found that, compared with other self-regulatory traits, grit focusing on long-term growth toward one’s higher potential is positively associated with PWB in university students (Vainio and Daukantaitë, 2016). As suggested in cultural values of Confucianism, self-regulatory process and perseverance emphasizing the positive value of adversity and people’s capacity to overcome difficulties are profoundly underscored (Shek, 2004). On the other side, under the influence of Buddhism, Tibetans are also determined to exert continuous efforts (mediation and doing charitable deeds) to lift the soul to reach external bliss (Lu, 2005). Due to the importance of perseverance and ambitions toward goals in both cultural contexts, we suggest that grit is appropriate for our research purposes, in comparison to other self-regulatory traits.

Few studies have focused on the antecedents of grit, however, available theory and empirical evidence support the positive association between PAS and grit to some extent. For example, based on SDT, autonomy support from parents is one of the crucial factors in promoting optimal development (Ryan and Deci, 2000), such as grit. An empirical study found that family relatedness is positively associated with high levels of grit (Datu, 2017), indicating that positive parenting and family relations may elevate the level of PWB. Studies have indicated that grit is related to increased academic performance and PWB in college students (e.g., Bazelais et al., 2016; Wolters and Hussain, 2015). Gritty individuals demonstrate consistent interests and work hard toward their goals with perseverance and these characteristics may enhance positive psychosocial adjustment in emerging adults. While it is documented that grit is positively related to PWB, much research is warranted to explore whether the association between grit and PWB may vary according to intercultural differences.

### The Present Study

**FIGURE 1:**
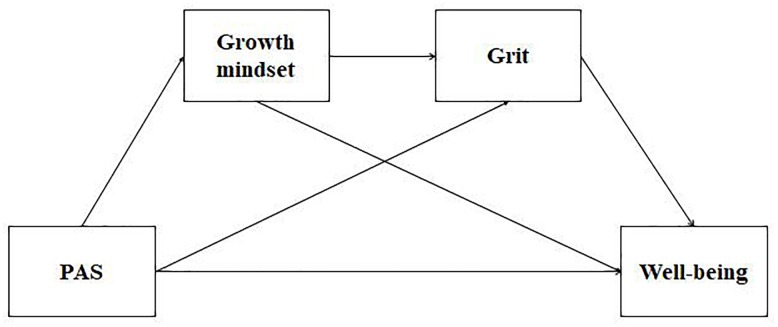
A hypothesized serial multiple mediation model. Age, gender, SES were considered as control variables. PAS, parental autonomy support.

The current study has three main goals. First, we examined the differences in the perception of PWB among Tibetan emerging adults, in comparison with their peers from the Han ethnic group. To our best knowledge, few studies to date have examined psychosocial outcomes in terms of Tibetans. No definite prediction was made concerning the differences in PWB between the two ethnic groups. Secondly, we explored the direct and indirect effects of PAS, growth mindset and grit on PWB in Tibetan and Han emerging adults. Based on the literature and theories reviewed above, we expected a serial multiple mediation model of growth mindset and grit in the association between PAS and PWB (see Figure 1). Third, we investigated whether these direct and indirect associations show similar patterns across the two ethnic groups. In line with intercultural differences between two ethnic groups, we hypothesized that these relationships varied between Tibetan and Han emerging adults.

## Materials and Methods

### Participants

The study participants comprised 59 Tibetan (71.2% girls) and 118 Han (69.5% girls) emerging adults aged between 18 and 25 years (*M* = 21.18; *SD* = 1.62), who were attending the minority college in Lanzhou, China. Based on national statistics, family SES for Tibetan and Han emerging adults was relatively moderate, with an average income equivalent to 500–800 United States dollars per month. In terms of parental education level, the majority of fathers (86.4%) and mothers (91.5%) of Tibetan emerging adults had finished primary and middle school and 55.9% of fathers and 67.8% of mothers of Han emerging adults had completed primary and middle school. The average length of education in Han cultural areas for Tibetans was approximately 3 years (range from 1 to 5 years, *SD* = 0.82).

Preliminary analyses indicated that the two ethnic groups did not differ in age (*t* = -0.95, *df* = 234, *p* = 0.34) or gender distribution (χ^2^ = 0.03, *df* = 1, *p* = 0.87), however, emerging adults from the Han ethnic group reported higher levels of SES than their peers from the Tibetan group (*t* = -2.82, *df* = 234, *p* < 0.01).

### Measures

#### Sociodemographic Characteristics

Demographic information collected from the participants included gender, age, place of birth, length of domicile in a Han cultural area, family composition, parental educational level and occupation and monthly family income. Socioeconomic status (SES) was assessed via maternal and paternal education background, occupation and family monthly income. There were four categories of parental education: (1) middle school graduation or lower, (2) high school graduation, (3) bachelor’s degree graduation and (4) master’s degree graduation or higher. Participants selected their parental occupations and monthly family income from among seven different options based on occupational classification and resident income criteria in China. The three scores were standardized and summed to yield an overall SES score (Seyfried, 1998).

#### Parental Autonomy Support

Parental autonomy support was measured using the Perceived Parental Autonomy Support Scale (P-PASS), initially developed for college students (Mageau et al., 2015). This 12-item instrument measured perceptions of three autonomy-supportive behaviors (choice, four items; rationale, four items; acknowledgment of feelings, four items; e.g., “My point of view was very important to my parents when they made important decisions concerning me”). Participants rated items in terms of how applicable each statement was to their relationship with their parents. Responses ranged from 1 (*do not agree at all*) to 7 (*very strongly agree*). The average score of all items was calculated, with higher scores indicating higher levels of perception of autonomy support from parents. Previously, this scale was used with a United States sample that included an Asian American population (Won and Yu, 2018), showing a good internal consistency. Cronbach’s alpha for this scale was 0.81 and 0.85 for Tibetan and Han emerging adults, respectively.

#### Growth Mindset

Growth mindset was measured using the six-item Implicit Theory Scale (Dweck et al., 1995). Originally, this scale consisted of four subscales (i.e., intelligence, morality, general world and personality). Due to methodological concerns raised in previous studies (e.g., Cheng and Hau, 2003), exploratory factor analysis (EFA) was adopted to examine reliability in the current study. The results supported the two factors, namely the general world and personality (χ^2^/*df* = 3.48, *p* < 0.001, CFI = 0.95, TLI = 0.90, SRMR = 0.04). One of the examples is “Though we can change some phenomena, it is unlikely that we can alter the core dispositions of our world.” Participants were required to rate each item based on a Likert scale ranging from 1 (*strongly agree*) to 6 (*strongly disagree*). An average score of six items was determined, with higher scores suggesting a stronger belief that the general world and personality can be changed. According to previous studies conducted in Chinese populations, this scale showed a satisfactory internal consistency (Wang et al., 2018). In the current study, Cronbach’s alpha was 0.80 and 0.82 for Tibetan and Han emerging adults, respectively.

#### Grit

Grit was measured by the eight-item Grit Scale-Short Form (Grit-S; Duckworth and Quinn, 2009), which was validated in Chinese cultures by Li et al. (2018). The scale consists of two dimensions: perseverance of effort (four items; e.g., “Setbacks do not discourage me”) and consistency of interests (four items; e.g., “New ideas and projects sometimes distract me from previous ones”). Participants were asked to rate each item from 1 (*not like me at all*) to 5 (*very much like me*) based on the Likert scale. The average score of eight items was calculated, with higher values indicating higher levels of grit. Previous studies reported the good internal consistency of this scale in a Chinese population (e.g., Li et al., 2018). In the current study, Cronbach’s alpha was 0.69 and 0.70 for Tibetan and Han emerging adults, respectively.

#### Psychological Well-Being

Psychological well-being was measured using the Flourishing Scale (FS; Diener et al., 2010), which was developed to measure PWB from an eudaimonic perspective. FS was validated in Chinese cultures by Tang et al. (2016). The scale is composed of eight items (e.g., “I lead a purposeful and meaningful life”). Participants were asked to rate each item from 1 (*strongly disagree*) to 5 (*strongly agree*) on the Likert scale. The average score of eight items was calculated so that higher scores indicated higher levels of PWB in essential aspects of the functioning and flourishing of respondents. Previous studies suggest the good internal consistency of this scale (Tang et al., 2016). In the current study, Cronbach’s alpha was 0.82 and 0.88 for Tibetan and Han emerging adults, respectively.

### Procedure

Ethical approval was obtained from the Ethics Review Board in the relevant university prior to data collection. Previous study (Yong, 2013) has suggested that most Tibetan emerging adults studying in the Han educational system may have difficulties with academic adaptation. Before the assessment, a trained researcher who was familiar with both languages examined the availability of Mandarin in Tibetan emerging adults, in order to make sure that the participants’ language proficiency was high and that they could understand all the measures well in Mandarin. To be specific, we randomly selected 20% items from each measurement in the current study and asked the participants to translate and back-translate (i.e., Mandarin-Tibetan and Tibetan-Mandarin) in front of a trained research assistant, informed by previous standardized procedures (Van de Vijver and Leung, 1997). Overall, 59 Tibetan emerging adults were qualified to participate in the current study.

Due to the small sample size, and suggestions proposed by recent studies that socio-demographics affect the perception of PWB (Frey and Stutzer, 2001; Pinquart and Sörensen, 2001), propensity score matching analysis (Randolph et al., 2014) was used to balance the two groups in terms of age, gender and SES. Based on the ratio of Han and Tibetan populations in the targeted province in China released by National Bureau of Statistics (2010), one young adult from Tibetan group was matched with two adults without any religious belief (e.g., Buddhism) from the Han ethnic group (for further details refer to Supplementary Materials).

Participants were recruited through a public college located in Lanzhou, China. After obtaining permission from the school principals, informed consent forms were given to each participant. After receiving confirmation of written consent, a trained researcher provided standardized instructions and the adults were asked to complete the online questionnaires during a 25-min period in the classroom. The first page of the questionnaire emphasized that participation was voluntary and anonymous. Upon completion of the surveys, the adults received a random cash reward ranging from 1 to 10 RMB from the online questionnaire system (*Wenjuan Wang*) to thank them for their participation.

## Results

### Preliminary Analyses

Data analyses were performed using R software (R Core Team, 2017). Before conducting the analyses, graphs (i.e., boxplot and Q–Q plot) were used to check normality and the presence of outliers in study variables. No relevant departure from normality assumptions and no extreme outliers were identified. No missing values were obtained in the current study as the targeted settings in the online questionnaire system did not allow questionnaires with missing values to be submitted.

Harman’s single-factor test was used to examine the effect of common method bias. The results showed that there were nine factors with the eigenvalue greater than 1 and the interpretation rate of the first factor was 23.82% (less than 40%; Podsakoff et al., 2003). Thus, no common method variance was found in the current study.

Means and standard deviations for study variables and bivariate correlations are reported in Table 1, separately for Tibetan and Han emerging adults.

**Table 1:** Descriptive statistics and bivariate correlations of study variables for Tibetan and Han emerging adults.

	Tibetan (*n* = 59)			Han (*n* = 118)									
	*M*	*SD*	Range	*M*	*SD*	Range	1	2	3	4	5	6	7
1. PAS	5.40	0.90	4–7	4.90	1.16	2–7	-	0.28**	0.16	0.23*	0.01	0.03	0.11
2. Growth mindset	2.61	0.59	1–5	2.58	0.71	1–5	0.19	-	0.15	0.02	-0.12	0.05	0.06
3. Grit	3.28	0.47	1–5	3.29	0.60	2–5	0.27*	0.01	-	0.52***	0.03	-0.06	0.16
4. Psychological well-being	5.27	0.75	4–7	5.05	0.97	2–7	0.44***	-0.04	0.36**	-	0.04	-0.02	0.29**
5. Age	21.54	1.61	18–25	21.00	1.60	18–25	0.08	0.15	0.08	-0.05	-	0.02	-0.16
6. Gender	-	-	1–2	-	-	1–2	-0.10	-0.07	0.15	-0.03	-0.28*	-	-0.01
7. SES	-1.09	1.62	–5.56	0.55	2.19	–2.93	0.25	0.06	-0.15	0.05	-0.23	0.00	-
			–2.73			–5.90							


*Correlation coefficients displayed above the diagonal are for adults from Han ethnic group, below for adults from Tibetan ethnic group. PAS, parental autonomy support; SES, socioeconomic status. ^∗^*p* < 0.05, ^∗∗^*p* < 0.01, ^∗∗∗^*p* < 0.001. Gender was coded as: 1 = male, 2 = female.*

As shown in Table 1, correlational analyses indicated that PAS and grit were each significantly and positively associated with PWB in Tibetan emerging adults. PAS was positively related to growth mindset and PWB in the Han ethnic group and grit was positively associated with PWB.

After controlling for age, gender and SES, and ANOVA was used to check the difference in PWB between two ethnic groups. The results showed that Tibetan emerging adults scored higher on PWB [*F*(1,172) = 6.49, *p* < 0.01, $\eta_{p}^{2}$ = 0.04] compared to their peers from the Han ethnic group, although the effect size was low. The effect of the period that Tibetan emerging adults had lived in Han cultural areas on PWB was also examined, and an ANOVA revealed that there was no significant difference as a function of the length of living in a Han cultural area [*F*(4,54) = 0.117, *p* = 0.976].

### Model Assessment

The small sample size prevented us from using a latent approach, which yield too many estimated parameters in relation to the number of participants assessed in this study. It should be noted that all measures (except PAS) were previously validated in Chinese cultural contexts (see section ‘Measures’ for more details), thus ensuring reliable estimates. For these reasons, we chose to apply a more parsimonious structural equation modeling (SEM) approach (i.e., path analyses) to evaluate the contributions of PAS, growth mindset and grit to PWB between Tibetan and Han emerging adults, using the package lavaan in R (Rosseel, 2012). A direct relationship between PAS and PWB was hypothesized and an indirect relationship between these two variables via the serial multiple mediating roles of growth mindset and grit was also investigated (see a hypothesized model at Figure 1). Gender, age and SES were considered as control variables, since previous studies had showed that these variables are associated with PWB. For example, Chinese girls show lower levels of well-being compared with boys (Ma et al., 2015). Chinese adolescents without economic disadvantage show higher levels of PWB, than their counterparts, Chinese adolescents with economic disadvantage (Shek, 2005).

First, multiple-group confirmatory factor analysis (MG-CFA) in lavaan was used to test measurement invariance in two ethnic groups (Hirschfeld and Von Brachel, 2014; Brown et al., 2017). The CFI difference (i.e., 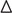
CFI) across the models was used to assess the overall path coefficients invariance instead of, which is quite sensitive to sample size (Cheung and Rensvold, 2002). The fully unconstrained model provided a good fit to the data [χ^2^ = 3.67 (6, *n* = 177), *p* = 0.72; NNFI = 1.09; CFI = 1.00; SRMR = 0.03], confirming the configural invariance of the model across the two ethnic groups. Accordingly, metric and scalar invariance was tested and the findings showed a non-significant *p*-value and no more than 0.01 of CFI difference, indicating that the hypothesized model was invariant across the two ethnic groups (see Table 2).

**Table 2:** Measurement invariance of a serial multiple mediation model across the two ethnic groups (*N* = 177).

Model	BIC	χ^2^	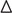 χ^2^	df	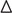 df	CFI	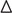 CFI	*p*
Configural invariance	1146	14.85	–	12	–	0.95	–	–
Metric invariance	1146	14.85	–	12	–	0.95	–	–
Scalar invariance	1132	17.17	2.31	15	3	0.97	0.01	0.50


**FIGURE 2:**
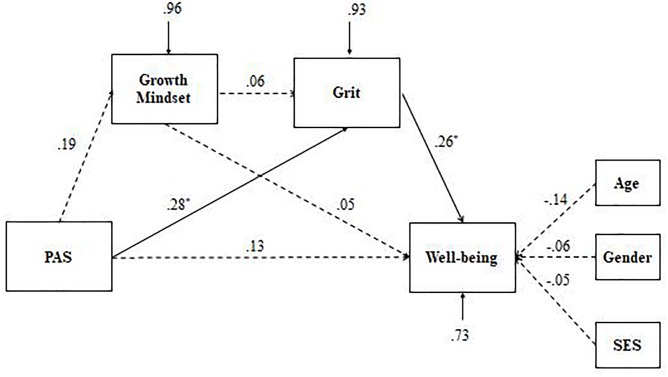
Path analytic models of the effects of parental autonomy support, growth mindset and grit on well-being in Tibetan emerging adults (*n* = 59). ^∗^*p* < 0.05. Dashed lines refer to non-significant association at a 0.05 level. PAS, parental autonomy support; SES, socioeconomic status.

**FIGURE 3:**
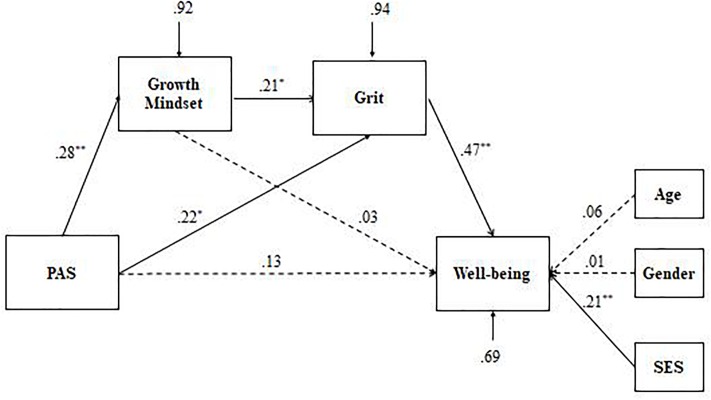
Path analytic models of the effects of parental autonomy support, growth mindset and grit on well-being in Han emerging adults (*n* = 118). ^∗^*p* < 0.05, ^∗∗^*p* < 0.01. Dashed lines refer to non-significant association at a 0.05 level. PAS, parental autonomy support; SES, socioeconomic status.

Next, multiple-group structural equation modeling (MG-SEM) in lavaan was used to examine the group differences in direct and indirect associations among study variables. A series of nested models was tested by constraining different paths as equal (Models 1–7, see Table 3). Suggested by previous studies (Marsh et al., 2004; Iacobucci, 2010; Kenny et al., 2015), absolute fit indices (e.g., NNFI, CFI, and RMSEA) are easily affected by small sample size. In the case of low *df* and small sample size, the SRMR is prioritized, where a value less than 0.08 is generally considered a good fit (Hu and Bentler, 1999). In terms of the equal value in the SRMR (Model 1, Model 3, and Model 5), NNFI and CFI was used to examine the equivalence of the model. Overall, comparison of the various models showed that Model 5, in which path coefficient from growth mindset to PWB was constrained to be equal, showed the best fit to the model, χ^2^(13) = 14.85, NNFI = 0.938, CFI = 0.973, SRMR = 0.045, indicating that the a proposed serial multiple mediation model varied across the two ethnic groups, especially focusing on the association between growth mindset and PWB. Further analyses (see Figure 2, 3) revealed that compared with the Han emerging adults, the relationships between PAS and growth mindset and between growth mindset and grit were not significant in Tibetan emerging adults.

**Table 3:** Tested models in multi-group analyses.

Model	χ^2^ (*df*)	NNFI	CFI	SRMR	*p*
Model 1	14.85 (12)	0.896	0.958	0.045	0.25
Model 2	15.42 (12)	0.919	0.965	0.047	0.28
Model 3	15.05 (13)	0.931	0.970	0.045	0.30
Model 4	16.44 (13)	0.884	950	0.049	0.23
**Model 5**	**14.85 (13)**	**0.938**	**0.973**	**0.045**	**0.31**
Model 6	17.58 (13)	0.846	0.933	0.048	0.17
Model 7	16.78 (13)	0.873	0.945	0.051	0.21


*Model 1: All path coefficients in the structural model were estimated freely. Model 2: Path coefficient from PAS to growth mindset was constrained to be equal. Model 3: Path coefficient from PAS to grit was constrained to be equal. Model 4: Path coefficient from growth mindset to grit was constrained to be equal. Model 5: Path coefficient from growth mindset to PWB was constrained to be equal. Model 6: Path coefficient from PAS to PWB was constrained to be equal. Model 7: Path coefficient from grit to PWB was constrained to be equal. The best fit model has been emboldened (Model 5)*.

As shown in Table 4, significant total indirect effects were observed (as confidence intervals did not contain a zero) in Tibetan and Han emerging adults. The indirect effect could be broken down into three unique effects via growth mindset and grit, independently and also in serial. Specifically, only the indirect effect via grit was supported for Tibetan emerging adults, whereas indirect effects via grit and both growth mindset and grit were confirmed for Han emerging adults. As no significant direct effect remained between PAS and PWB once the mediators were included in the model, it can be concluded that the relationship between PAS and PWB is fully mediated in both Tibetan and Han emerging adults.

**Table 4:** Indirect effects based on 5,000 bias-corrected bootstrapped samples.

Model	Standardized probit effect	Boot *SE*	Boot LLCI	Boot ULCI
Tibetan emerging adults (*n* = 59)				
Total indirect effect: PAS-PWB	0.062	0.081	0.061	0.243
Indirect effect via growth mindset	-0.011	0.046	-0.093	0.082
Indirect effect via grit	0.078	0.056	0.002	0.219
Indirect effect via both growth mindset and grit	-0.005	0.016	-0.069	0.005
Han emerging adults (*n* = 118)				
Total indirect effect: PAS-PWB	0.060	0.049	0.024	0.173
Indirect effect via growth mindset	0.006	0.024	-0.033	0.066
Indirect effect via grit	0.078	0.044	0.001	0.174
Indirect effect via both growth mindset and grit	-0.024	0.014	-0.067	-0.004


*PAS, parental autonomy support; PWB, psychological well-being*.

## Discussion

The main goal of the current study was to compare PWB between Tibetan and Han emerging adults. While extant research suggests that social contexts and individual characteristics contribute to PWB, little is known about whether autonomy support from parents is beneficial to PWB in a collective setting. The underlying mechanism between PAS and PWB is also still underexplored. Our results showed that Tibetan emerging adults perceived higher levels of PWB than their peers from the Han ethnic group. Importantly, a serial multiple mediation model was supported in Han emerging adults, wherein PAS was associated with greater growth mindset, which in turn was associated with greater grit, and then with higher PWB. The indirect effects in both ethnic groups varied. Grit fully mediated the link between PAS and PWB in both ethnic groups, whereas the mediation role of growth mindset showed a significant indirect effect in the Han ethnic group only.

Our first purpose was to compare PWB between Tibetan and Han emerging adults. The findings showed that Tibetan emerging adults perceived higher levels of PWB than their counterparts from the Han ethnic group, confirming that intercultural differences may lead to the different perceptions of PWB from a culture and well-being perspective. One possible explanation align with the different basis for cultural values between Tibetan and Han emerging adults. Compared with the Han ethnic group who are greatly influenced by Confucianism, Tibetan emerging adults grounded in Buddhism find it much easier to fulfill their subjective happiness because religious and cultural practices emphasize the cultivation of mental balance and reflections into the nature of reality. Additionally, due to the geographical differences, Han areas are strongly influenced by Western cultural values, such as utilitarianism and materialism. As suggested by previous studies (e.g., Sachs et al., 2018), wealth is treated as one of the main indicators with which to evaluate the level of PWB. As such, competitions for higher income and standard of living in Han cultural areas may exacerbate the subjective evaluation of happiness. Our current findings also showed that higher SES was positively associated with the PWB of emerging Han adults, but not for Tibetans, which also supports this justification to some extent.

A serial multiple mediating role of growth mindset and grit was confirmed in the link between PAS and PWB in Han emerging adults. Congruent with SDT and previous research (Ryan and Deci, 2000; Kim et al., 2017), PAS is beneficial to developing optimal functioning, such as growth mindset and grit, which in turn enhance well-being for emerging adults. One possible explanation is that as autonomy needs expand during emerging adulthood, PAS can promote the inherent growth of emerging adults to convince them that basic attributes are malleable and developable through sustained efforts. When encountering challenging circumstances, emerging adults who still commit to a long-term goal orientation may perceive a positive emotional and cognitive evaluation of their life. In the meanwhile, the current findings did not show a significant direct effect between PAS and PWB: the influence of social contexts on the psychosocial functioning of emerging adults may play a role through personal characteristics.

A multi-group comparison and further investigation by each ethnic group separately shed light on the different indirect effects in the association between PAS and PWB in Tibetan and Han emerging adults. The current study failed to find a significant indirect effect of the growth mindset in Tibetan emerging adults. One possible explanation is that this may align with cultural values in Buddhism emphasizing transmigration and acceptance instead of active coping. As such, whether selfhood and the general world can be changed is determined by spirituality but not personal potentials. Moreover, suggested by prior research (Snibbe and Markus, 2005), comparing with adults from higher SES backgrounds, individuals with lower SES show lower levels of agency. In the current study, compared with Han emerging adults, Tibetans showed relatively lower levels of SES, which can also explain the lower levels of human agency. Another explanation is ascribed to the small sample size used for Tibetan emerging adults. Prior study has revealed that fit indices are sensitive to sample size (Cheung and Rensvold, 2002), which may lead to a non-significant indirect effect of growth mindset. Future studies using a relatively larger sample size are warranted to confirm the current findings.

### Limitations and Implications

While this exploratory study adds to the extant literature by pointing out the perception of the differences in PWB between Tibetan and Han emerging adults, and documenting the serial mediating roles of growth mindset and grit in the association between PAS and PWB, a number of limitations should be considered. First, the cross-sectional design did not rule out the directionality of the current findings. Although, the current study is guided by a self-determination framework and implicit theory, as well as supported by empirical evidence positing that high levels of growth mindset affect grit, it could not rule out whether grit predicts growth mindset in Tibetan and Han emerging adults. Future studies using a longitudinal design are warranted to check the current findings. Second, Tibetan emerging adults in the current study were recruited from an ethnic minority university located in Gansu Province, which generally comprises Tibetan settlements. While the duration of living in a Han cultural area was also considered, the effects of migration and the differences in SES between home and destination locations on PWB in emerging adults could not be ruled out. Future studies may consider the location of sampling to better generalize the current findings. Third, while a number of statistical strategies were used, the inability to detect significant effects in either direct or indirect relations may be due to the relatively small sample size, especially for Tibetan emerging adults. Future studies with a larger sample size should be carried out to explore the targeted associations. Fourth, both Tibetan and Han emerging adults were questioned in Mandarin. While language proficiency in Mandarin was examined, the use of Mandarin may be a limitation when interpreting the findings for Tibetan emerging adults. Future studies are strongly encouraged to use the Tibetan native language to check the current findings. The self-report measurements in the current study have also not been previously validated in Tibetan cultures, which may also lead to inaccurate results. Indeed, self-report measurement also limited the validity of the current findings. Future studies using a mixed-method approach are necessary to examine the current results.

## Conclusion

To conclude, the present study suggests that Tibetan emerging adults perceive a high level of PWB compared to their peers from the Han ethnic group. Meanwhile, PAS enhances the level of PWB through the serial multiple mediating roles of growth mindset and grit in Han emerging adults. Han emerging adults may benefit from targeted school activities or intervention programs aimed to elevate growth mindset and grit. Educators and practitioners could design activities focusing on targeted challenging goals, in order to encourage emerging adults to achieve their goals through persist supervision, group cooperation and continuous practice, as well as to convince emerging adults that the basic attributes can be improved or changed continuous efforts. In the face of adversities, achieving long-term goals are prioritized. Autonomy support from parents is critical in both cultural contexts, however, intervention focusing on elevating grit, but not growth mindset, may have a better effect in enhancing a positive emotional and cognitive evaluation of quality of life in Tibetan emerging adults.

## Data Availability

The datasets for this manuscript are not publicly available because dataset concerning ethnic minority information is sensitive to publish. Requests to access the datasets should be directed to lanxiaoyu1001@163.com.

## Author Contributions

XL analyzed the data and drafted the manuscript. CM supported for the data collection and reviewed the manuscript critically. RR assisted in the preparation of the manuscript and data management. All authors have made a substantial, direct and intellectual contribution to the work, and approved it for publication.

## Conflict of Interest Statement

The authors declare that the research was conducted in the absence of any commercial or financial relationships that could be construed as a potential conflict of interest.
