# Supplementary material for: Parental Autonomy Support and Psychological Well-Being in Tibetan and Han Emerging Adults: A Serial Multiple Mediation Model
Source: Front Psychol. 2019 Mar 21;10:621. doi: 10.3389/fpsyg.2019.00621 (PMC6437062; doi:10.3389/fpsyg.2019.00621)
Supplement: Supplementary file 1 [file Table_1.DOCX]

**Appendix A**

Propensity scoring analyses in R using package MatchIt (Randolph, Falbe, Manuel, & Balloun, 2014) was used to match the two ethnic groups regarding age, gender, socioeconomic status. Nearest neighbor method was used to compare the two ethnic groups (Cover & Hart, 1967) and one-to-two matching ratio was adopted. Namely, one emerging adult from Tibetan group was matched with two emerging adults from Han ethnic group.

The results show that matching fits well in the current study. According to jitter plot where each circle represents a case’s propensity score (see Figure 1), the absence of cases in the uppermost stratification indicates that there are no unmatched subjects from Tibetan group. The middle stratification shows the close matching between the Tibetan group and the Han ethnic group. The downmost stratification shows the unmatched units from Han ethnic group, which are not used in any follow-up analyses. Moreover, histograms plot shows that the distribution of Tibetan and Han ethnic group is quite similar (see FIGURE 2). In sum, the visualized figures show that the matching is acceptable. The final analytical sample consists of 177 emerging adults, with 59 adults from Tibetan group and 118 adults from Han ethnic group.


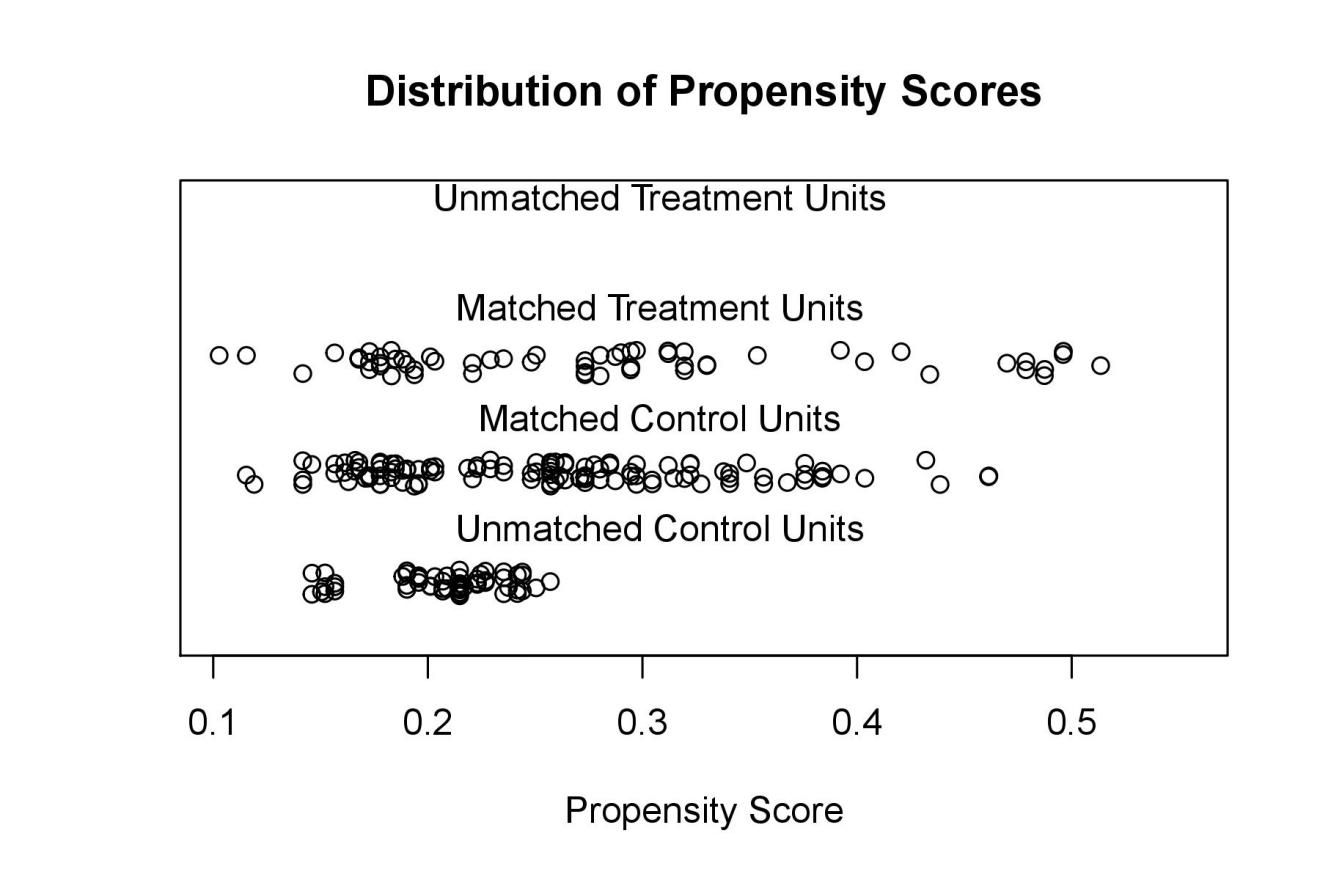


**FIGURE 1** Jitter plot for matching the Tibetan group and the Han ethnic group.

*Note.* Treatment units = Tibetan group, Control units = Han ethnic group.


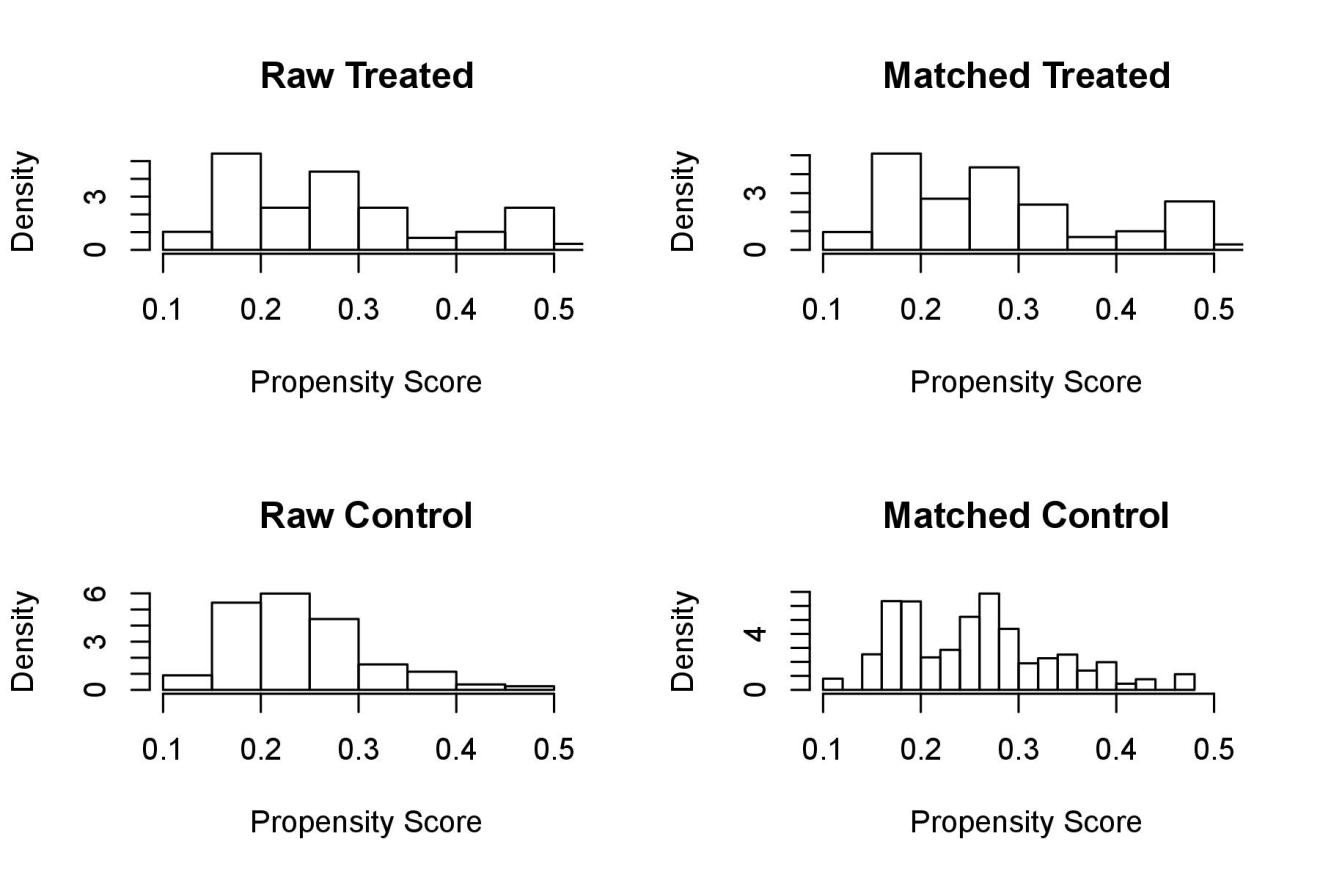


**FIGURE 2** Histogram plot for matching the Tibetan group and the Han ethnic group.

*Note.* Raw Treated = Raw Tibetan group, Raw Control = Raw Han ethnic group, Matched Treated = Matched Tibetan group, Matched Control = Matched Han ethnic group.
